# Measurement of trainee autonomy in robotic-assisted lung resections using objective performance indicators

**DOI:** 10.1007/s11701-026-03622-z

**Published:** 2026-10-05

**Authors:** Aaron Guo, Jad Elharake, Chandler Lowe, Mahmoud Abdel-Rasoul, Marissa Guo, Shi-Hao Lee, Mallory Shields, Daniel Oh, Ioana Baiu, Peter J. Kneuertz, Robert E. Merritt, Desmond M. D’Souza

**Affiliations:** 1 Division of Thoracic Surgery, Department of Surgery, The Ohio State University Wexner Medical Center, 410 10th Avenue, N835 Doan Hall, Columbus, OH 43210 USA; 2 Center for Biostatistics, Department of Biomedical Informatics, The Ohio State University Wexner Medical Center, Columbus, OH USA; 3 Research Division, Intuitive Surgical Inc., Sunnyvale, CA USA

**Keywords:** Robotic-assisted thoracic surgery, Objective performance indicator, Trainee autonomy, Surgical education

## Abstract

Trainee autonomy in robotic-assisted thoracic surgery is critical for growth but difficult to quantify. Robotic platforms can log intraoperative kinematic and event data that can be transformed into metrics known as objective performance indicators (OPIs). This study sought to use OPIs obtained during robotic-assisted anatomic lung resections to assess trainee console autonomy at a high-volume robotic center. OPI data were obtained from 59 lobectomies and segmentectomies performed on a multi-port robotic platform between March 2022 and May 2023. Cases were divided into anatomic steps and annotated by a team of verified video reviewers. Console autonomy was assessed based on average path length (PL) of controllers and active time (AT) on the trainee console as a ratio compared to the overall PL and AT of the operation. Cases included 51 (86%) lobectomies and 8 (13%) segmentectomies. Average overall PL ratio was 0.15 (SD 0.17) and overall AT ratio was 0.23 (SD 0.23) for senior trainees. Autonomy was highest for inferior pulmonary ligament division (PL ratio = 0.39, AT ratio = 0.42) while lowest with pulmonary artery dissection (PL ratio = 0.10, AT ratio = 0.14). There was a significant association between AT and PL ratio with estimated blood loss (PL p = 0.036, AT p = 0.047). AT and PL ratios can provide objective surrogates of autonomy in thoracic surgery and can be stratified by individual case components. OPIs will offer valuable metrics for surgical education and assessment as national accrediting bodies shift graduation requirements towards case component proficiency over volume.

**Supplementary Information:**

The online version contains supplementary material available at https://doi.org/10.1007/s11701-026-03622-z.

## Introduction

It is well established that minimally invasive approaches to lung resections have improved outcomes and lower morbidity compared to open thoracotomy [1–6]. Since 2019, robotic-assisted operations have become the predominant approach to lobectomy and segmentectomy in the United States [7]. Unfortunately, robotic-assisted operations are associated with an arduous technical learning curve [8] and significant variation in exposure during training, making it difficult to design standardized curriculum for robotic skills. Most recently, the Society of Thoracic Surgeons (STS) published consensus recommendations on standardizing robotic surgery training for thoracic surgical trainees [9], including an emphasis on procedural milestones and graduated autonomy [9].

While these guidelines offer a scaffold for robotic thoracic training, operative autonomy for residents remains a challenging topic. Previous studies have noted challenges in balancing training opportunities with operating time, costs, and perioperative outcomes [10], particularly at centers where attending surgeons may be learning the robotic platform alongside their trainees. Assessments of autonomy are furthermore constrained by subjective measurements, with sometimes significant differences between attending versus resident reporting [11–13]. With a shift in surgical training towards clinical mastery along case-specific components, rather than pure case volume, there is growing need for better methods to evaluate fulfillment of said components.

A valuable but less acknowledged advantage of robotic platforms is the digitalization of most actions performed on the console. The Da Vinci Robotic Surgical System (Intuitive Surgical, Sunnyvale, CA) records data streams including operative videos, arm movement characteristics, and instrument action events, which can be analyzed to offer potential objective metrics for surgical performance. Objective performance indicators (OPIs) in thoracic surgery have been correlated with intraoperative complications and surgical experience [14–16]. Here, we explored the use of OPIs as a measurement of trainee autonomy in robotic-assisted lung resections in a real-world application.

## Methods

### Data collection

A total of 82 robotic-assisted lung resections were performed on the da Vinci Xi platform (Intuitive, Sunnyvale, CA) between March 2022 and May 2023 at a tertiary academic center. Of these, 22 cases were excluded: 16 wedge resections, due to an inherent difference in technical requirement; 4 cases which did not have a trainee present; 2 in which the case was aborted. A total of 59 robotic-assisted lobectomies and segmentectomies were included in the final analysis. The study was conducted in alignment with institutional ethical principles after approval by the Institutional Review Board (OSU IRB 2024H0268). Informed consent was not obtained due to the retrospective nature of the study.

### Setup and data source

All operations were performed on a dual-console arrangement with an attending surgeon and surgical trainee. Operations were performed by three senior thoracic surgeons who had completed their learning curve in robotic-assisted lung resections. Trainees on the thoracic surgery service included PGY 1–3 junior residents and PGY 6–7 senior residents and fellows. Backgrounds included integrated thoracic residents, general surgery residents, traditional thoracic surgery fellows, and acute care surgery fellows (Supplemental Table 1). All trainees were required to obtain the da Vinci System Training Certificate, complete 10 cases as the bedside assistant, and score a passing grade on a 13-module curriculum on the robotic simulator, prior to sitting at the console. Residents also participated in wet-lab robotic sessions as part of surgical didactics throughout the academic year. Robotic console data streams were collected using an Intuitive Data Recorder (IDR, Intuitive, Sunnyvale, CA) and included both event variables and kinematic variables. Event variables included clutch events, energy activations, arm swaps, and camera movements, whereas kinematic variables included arm movement in space, acceleration, pitch, roll, and yaw. Intraoperative videos from the robotic endoscope were also recorded for each case.

### Objective performance indexing

Each case video was reviewed by a team of certified surgical annotators using a standardized template for operative steps in robotic-assisted lobectomy. Components included inferior pulmonary ligament division, lymph node dissection, pulmonary vein dissection, fissure dissection, pulmonary artery dissection, pulmonary vein division, pulmonary artery division, bronchus dissection, and bronchus division. Each step was tagged by a start and stop time within the overall video, then associated with timestamped data from the IDR. Variables for components with multiple events, such as lymph node dissection, were combined or averaged when appropriate. OPIs were calculated from kinematic and event data for each step using proprietary algorithms from Intuitive (Sunnyvale, CA). Specific OPIs of interest included path length (PL), which describes the cumulative distance traveled by all the robotic arms, and active time (AT), which describes the cumulative time spent using the instruments. These OPIs have been previously demonstrated as a marker of console autonomy in other specialties [17, 18].

### Statistical analysis

Data were presented as mean ± standard deviation (SD) for continuous variables and counts with percentage (%) for categorical variables. OPIs including AT and PL were calculated for both trainee and surgeon console. A ratio for the trainee was generated by dividing the trainee AT and PL by AT and PL for the overall case, respectively. Ownership of the case was defined as AT or PL ratio > 0.5, indicating performance of at least half the operation by the trainee. Categorical variables were compared via Chi-square or Fisher exact test when appropriate. Continuous variables were compared with Student t-test and ANOVA. Univariable linear regression models were fit to assess correlation between each of AT and PL ratio with continuous perioperative outcomes. *P* < 0.05 was considered statistically significant for all tests. Statistical analyses were performed using SAS Version 9.4 (SAS Institute, Cary, NC) and RStudio Version 4.4.0.

## Results

**Fig. 1:**
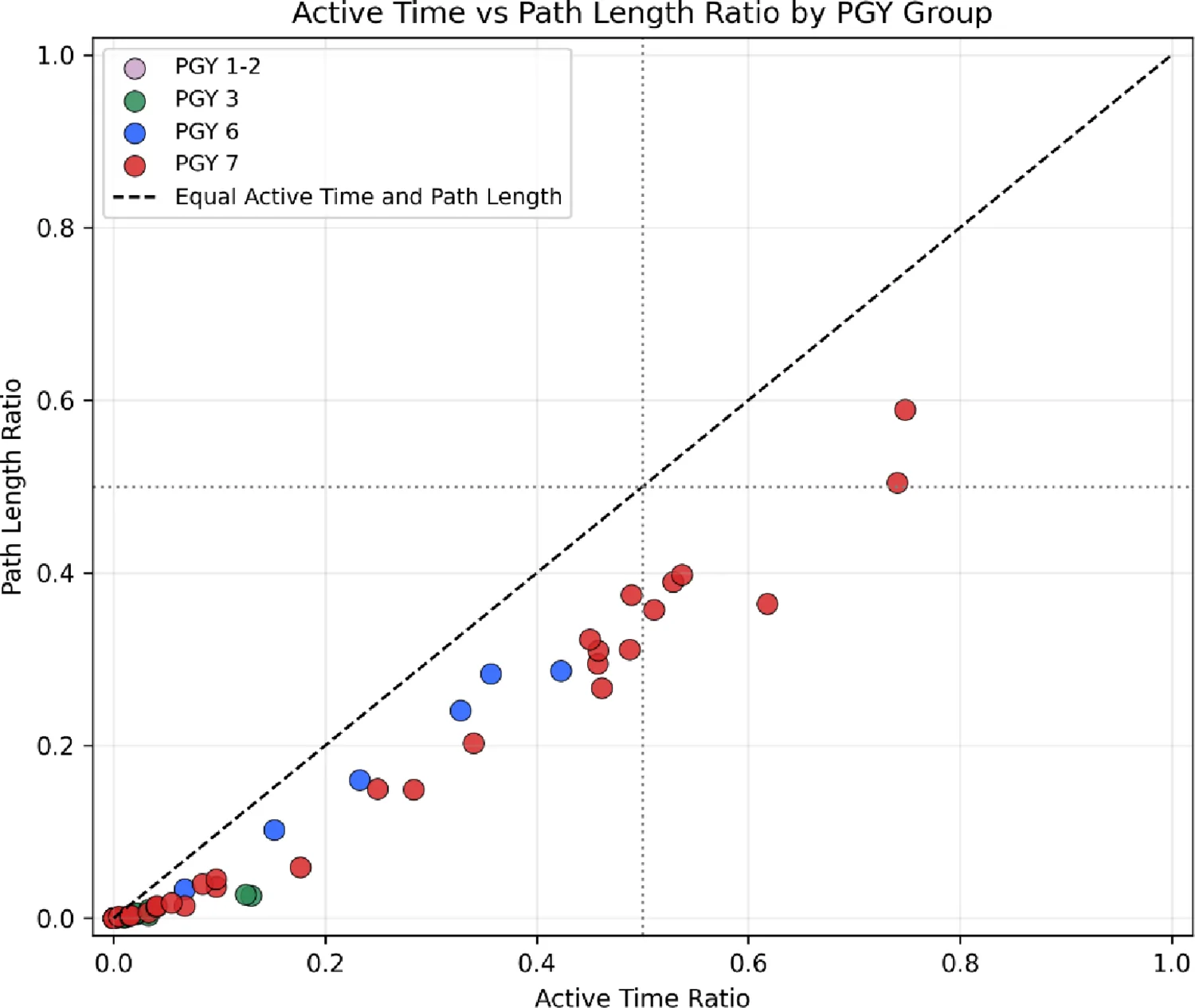
Distribution of active time and path length ratios by individual case

There were 51 lobectomies (86%) and 8 segmentectomies performed (14%) captured in the study period. Baseline patient demographics are listed in Table 1. 17 cases (29%) were performed with junior trainees versus 42 (71%) cases with seniors. There were 18 individual trainees including 10 junior and 8 senior residents. Mean console PL ratio for juniors was 0 (SD 0.01) vs. 0.15 (SD 0.17) for seniors, *p* < 0.001. Mean AT ratio for juniors was 0.02 (SD 0.04) vs. 0.23 (SD 0.23) for seniors, *p* < 0.001 **(**Fig. 1). For all trainees, there was no difference in autonomy based on lobectomy versus segmentectomy (PL 0.11 vs. 0.10, *p* = 0.8; AT 0.17 vs. 0.17, *p* > 0.9).

**Table 1:** Demographics

Comorbidities	mean ± SD or *n* (%)
Age (years)	65 ± 11
Male sex	25 (42%)
BMI (kg/m2)	29 ± 6
**Smoking status**	
Current Smoker	9 (15%)
Previous Smoker	36 (61%)
Never Smoker	14 (24%)
Diabetes Mellitus	15 (25%)
Chronic Obstructive Pulmonary Disease	27 (46%)
Coronary Artery Disease	14 (24%)
Peripheral Vascular Disease	6 (10%)
Hypertension	37 (63%)
ASA score	3 ± 0.3
Previous Thoracic Operation	29 (49%)
FEV1 (%)	83 ± 16

ASA score = American Society of Anesthesiologists Physical Status Classification. BMI = body mass index. FEV1 = forced expiratory volume in 1 s

**Fig. 2:**
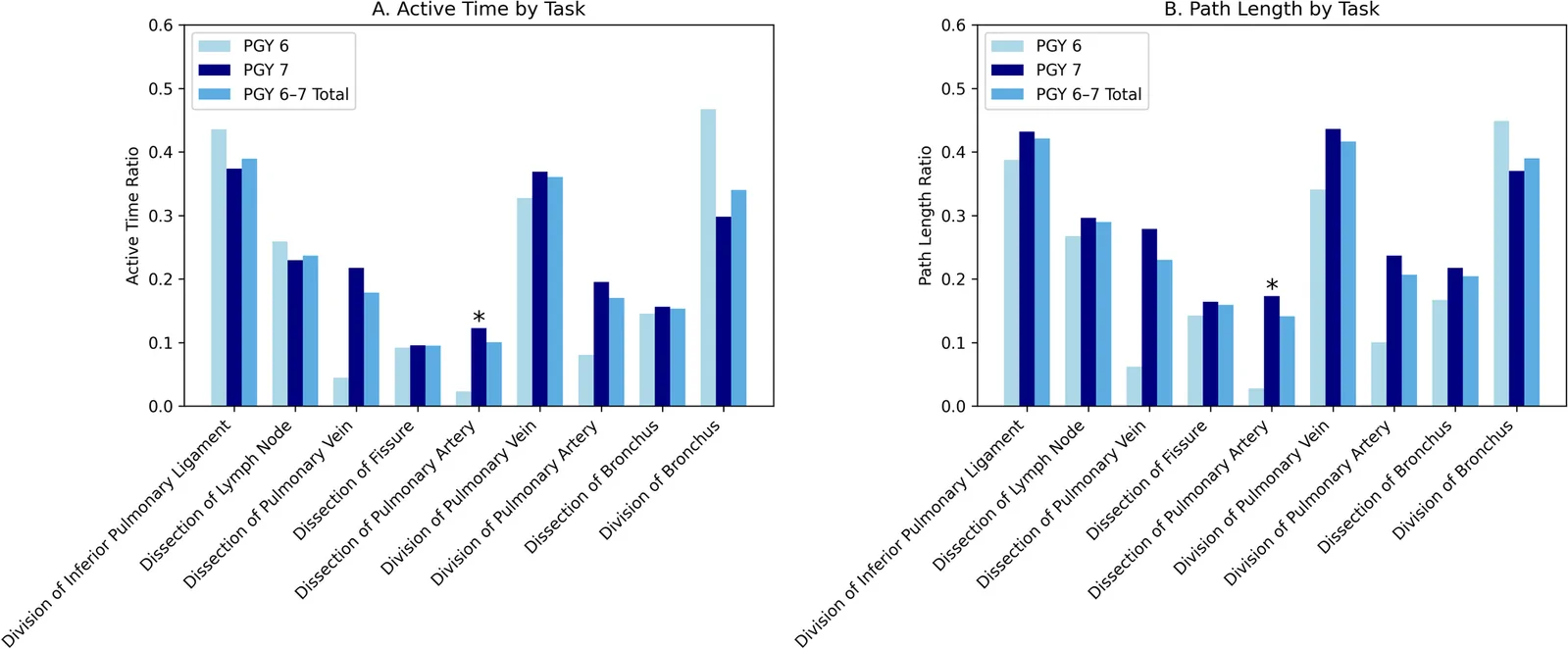
Comparison of autonomy between senior trainees for individual procedural tasks. within anatomic lung resections. Asterisk represents a statistically significant difference between training years (*p* < 0.05)

A further analysis of the senior trainees (PGY-6 and PGY-7) was performed. Ownership of the case, defined as > 0.5 PL or AT, for senior trainees was *n* = 2 (5%) and *n* = 6 (14%), respectively. In comparing senior trainees by PGY-6 (*n* = 10) versus PGY-7 (*n* = 32), there was no significant difference in autonomy for the overall case by PL ratio (*p* = 0.3) or AT ratio (*p* = 0.2). When broken down by individual procedural steps, there was a significant difference in autonomy between steps (*p* < 0.001), with the most autonomy in inferior pulmonary ligament division (PL 0.39, AT 0.42), and least autonomy in dissection of the pulmonary artery (PL 0.10, AT 0.14) (Fig. 2). Autonomy between PGY-6 versus PGY-7 residents was similar for each step, except for dissection of pulmonary artery, (PL 0.02 vs. 0.12, *p* = 0.025; AT 0.03 vs. 0.17, *p* = 0.018).

Perioperative outcomes are listed in Table 2. In a univariable regression, there was no significant correlation between either AT or PL ratio with operative time (PL *p* = 0.28, AT *p* = 0.25), operative cost (PL *p* = 0.08, AT *p* = 0.07), chest tube duration (PL *p* = 0.95, AT *p* = 0.99), or length of stay (PL *p* = 0.74, AT *p* = 0.64). AT and PL ratio were both associated with an increase in estimated blood loss (EBL) (PL *p* = 0.036, AT *p* = 0.047) (Supplemental Table 2).

**Table 2:** Perioperative Outcomes

Intraoperative outcomes	mean ± SD or *n* (%)
Estimated blood loss (mL)	32.5 ± 21.7
Operative time (min)	171.1 ± 48.5
Procedure Cost ($USD)	102,085.31 ± 20,667.87
**Post-operative outcomes**	
Chest tube duration (days)	2.9 ± 3.4
Length of stay (days)	3.1 ± 2
Narcotic use score (MME)*	94 ± 157.3
Mortality	0 (0%)

MME = morphine milligram equivalents. *Cumulative MME during post-operative admission

## Discussion

This study explored the use of OPIs to assess console autonomy in robotic-assisted thoracic surgery. Autonomy measured by AT and PL ratio were overall low but varied by trainee level. Senior residents demonstrated higher autonomy compared to juniors, performing on average 15% of the operation by PL ratio, and 23% of the case by AT ratio. There was a significant difference in autonomy based on case components, which correlated to technical demand and risk. In a limited analysis comparing the PGY-6 against PGY-7 trainees, we observed a statistically significant increase in autonomy based on training year only for pulmonary artery dissection. This finding met clinical expectations as senior fellows developed greater experience with the anatomy and the robotic console during their training; simultaneously, attendings may have entrusted more of this high-risk step to the senior trainees after building confidence on simpler procedural components. Interestingly, we did not observe a difference in autonomy between lobectomy and segmentectomies, perhaps due to a redistribution of autonomy to other steps that was not statistically detectable.

**Fig. 3:**
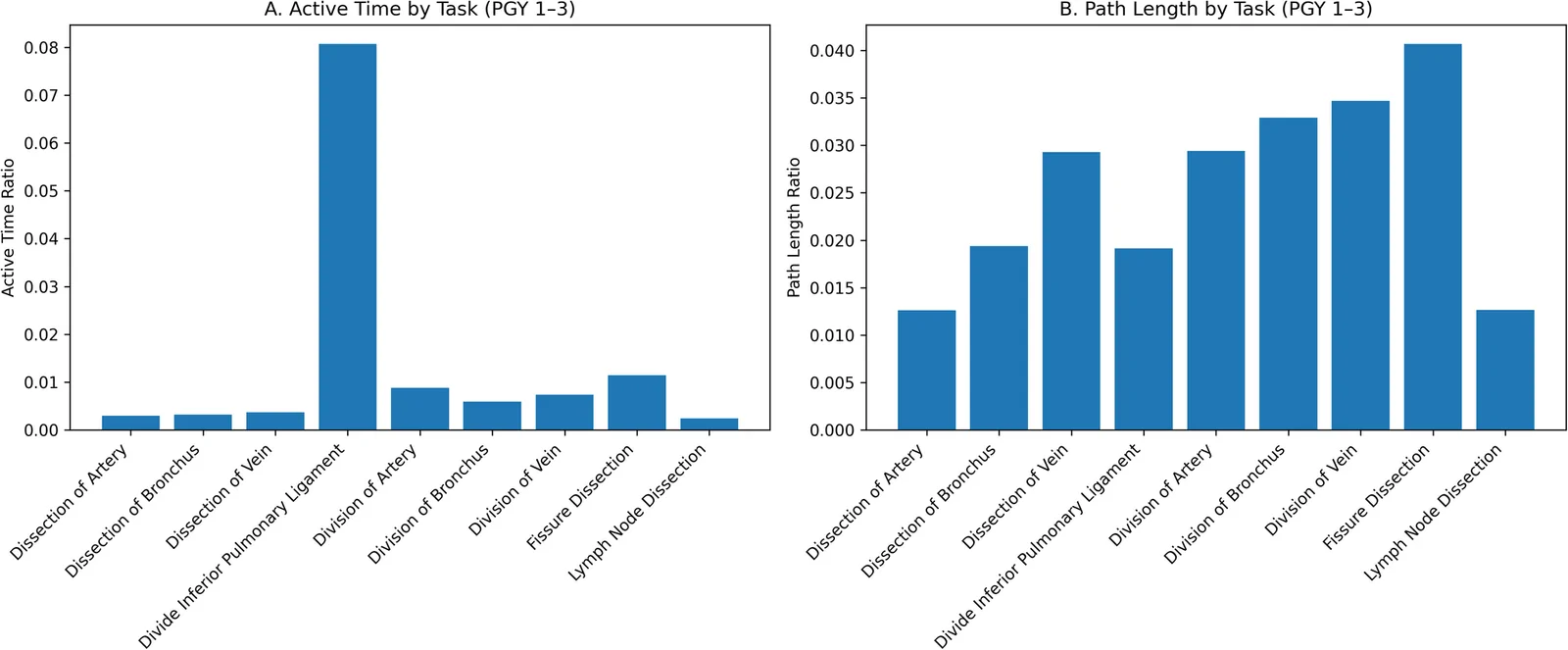
Autonomy within individual procedural tasks amongst junior trainees

As expected due to the significant risks and learning curve inherent to thoracic surgery, junior-level residents had minimal console participation with a modest amount of autonomy offered in simpler steps like pulmonary ligament division (PL = 0.08, AT = 0.02) (Fig. 3). However, the low levels of ownership and AT/PL ratios for senior residents raised significant concern. One potential explanation is the presence of four acute care surgery fellows, who were performing electives on thoracic surgery during the study period and were included in the senior resident cohort alongside four dedicated thoracic surgery fellows. These elective rotators were likely entrusted with less console activity which may have negatively skewed the overall distribution. Additionally, robotic-assisted wedge resections, which are more accessible to trainees, were intentionally excluded due to a difference in procedural components. Nevertheless, this important finding highlights the blind spots in surgical education, and the value of OPIs in providing an objective signal for the need to change. Since internal review of these data, there has been an anecdotal shift in training culture to improve trainee experience. Tools driven by artificial intelligence, such as Case Insights on the Da Vinci 5 (Intuitive, Sunnyvale, CA), can collect OPI data and console utilization rates in real-time after each case, and will empower us to objectively review this trend on a scheduled basis.

OPIs have demonstrated markers of limited autonomy in general surgical training as well [18]. Indeed, the trend of diminished autonomy for thoracic fellows observed here may be partly a downstream result of restricted autonomy or limited exposure in general surgical training. Graduation prerequisites for general surgery training set by the Accreditation Council for Graduate Medical Education (ACGME) include a vague requirement of any 20 thoracic operations, including 5 thoracotomies; a study of general surgery resident case logs from Regalie et al. indicated a significant decrease in the quality and quantity of exposure to thoracic surgery for trainees in recent years, particularly for chief-level residents who may not rotate on thoracic services at all [19]. Previous work by Yu et al. analyzed the Veterans Affair Surgical Quality Improvement Program database, identifying up to a 75% decrease in operative autonomy for general surgery trainees over the last two decades [20]. Furthermore, a separate study by Anjaria et al. using the same database noted severe decreases in resident ownership over time across most other fellowship-level subspecialties. The lowest proportion of resident-primary operations was in thoracic surgery, which decreased from a meager 3.7% in 2004, to just 0.5% by 2019 [21]. It is worth noting that this time coincided with the introduction of robotic-assisted surgery to the thoracic field, which likely had deleterious consequences for trainees in an already constrained environment. Finally, with public recording at large academic centers, faculty may be disinclined to relegate difficult procedures to trainees in order to limit the risk of adverse events.

In a limited analysis of procedural outcomes, there was not a statistically significant association between trainee autonomy and overall procedural cost or duration, contrasting with concerns from previous literature on robotic cholecystectomy and hernia repair [10]. However, trainee autonomy was associated with a small but statistically significant increase in EBL, with between 2-3mL increase per every 1% in AT or PL ratio. These findings provide a clinical correlation to previous work by Dr. Lazar et al.; in their study of 46 trainees operating on a perfused ex-vivo lobectomy model, specific OPIs were associated with bleeding events that occurred in 24% of procedures, defined as blood loss that interrupted the operation or required manual pressure or repair [16]. Given the overall modest blood loss across our cohort averaging 30 mL, the increased blood loss associated with trainee autonomy likely represented minor bleeding due to poor tissue handling or hemostasis in parenchymal and nodal dissection, rather than catastrophic events during vascular dissection. It is also plausible that due to the subjective nature of EBL documentation, dictations may have been biased towards reporting higher estimates in operations with more trainee participation.

Due to the resources needed to annotate each case video, we were unable to stratify autonomy by case complexity, which in turn limited analysis of perioperative outcomes. Further studies with multi-center data will help delineate the relative importance of certain OPIs in patient outcomes, and better define correlations to surgical efficiency. Within this domain, there is significant activity in using artificial intelligence to autonomously annotate videos immediately after they are recorded [22, 23]. Harnessing these tools and applying them within interfaces like the Intuitive Hub (Intuitive, Sunnyvale, CA) will exponentially improve case review, empower data collection, and facilitate personalized analysis of OPIs for training program curricula. By streamlining the review process, artificial intelligence will improve surgical training and facilitate analysis of adverse events, overall enhancing patient safety.

Although robotic-assisted approaches have steadily grown to become the pre-eminent modality of routine thoracic surgery over the last decade [7, 24], there remains a significant gap in surgical training. In a 2016 survey by the STS, over half (56%) of new graduates from cardiothoracic surgery training programs felt they lacked confidence or needed more instruction in robotic-assisted pulmonary resections [25]. In response, an STS task force was designated to design a formal curriculum on robotic-assisted thoracic surgery training. In their recently published guidelines, Kim et al. provided recommendations with support structures such as wet labs, dedicated bedside assistants, and dual-console arrangements to facilitate a training environment. The authors also emphasized case-specific procedural milestones, an eventual shift towards Entrustable Professional Activities (EPAs), and objective methods of feedback and assessment [9]. Whereas current ACGME graduation requirements for thoracic surgical trainees are based on case volume, EPAs would mandate demonstration of proficiency and appropriate supervision along operative milestones.

Traditional methods of surgical assessment, such as the Global Evaluative Assessment of Robotic Surgery, are performed manually. These methods are inherently limited by the skilled time required to review videos and by subjectivity between reviewers [11, 15]. OPIs could help bridge these shortcomings with objective, quantifiable metrics that can be abstracted autonomously from the robotic console. For example, ownership of the task by a specific PL ratio threshold could help validate actual performance of a milestone within the operation. Furthermore, measurements of AT ratio could ensure that trainees are being afforded ample opportunity to demonstrate their ability or to develop necessary skills. It is unclear what these thresholds for active time or ownership would be, as more experienced surgeons may complete tasks faster or incur shorter total path length due to better economy of motion; understanding these metrics will certainly be an area of interest in the future. Finally, previous studies have identified differences between OPI profiles of novice versus expert robotic thoracic surgeons, in both simulated and clinical experiences [16, 26, 27]. These OPIs, such as camera clutching, wrist articulation, bimanual symmetry, arm movement speed and smoothness, and idle time [16, 26], may offer focused areas for assessment and tangible targets for trainees. Future prospective cohort studies regarding trainee experience and measuring OPIs longitudinally will characterize how robotic skills mature over time and offer additional insight as to optimal development pathways.

### Limitations

Due to small sample size and variation in oncologic presentation and operative anatomy, controlling for case complexity was limited, which in turn curtailed further analysis of outcomes. There were also certain trainees, particularly traditional-track thoracic fellows, who participated throughout the two-year pilot study period. These trainees likely experienced progressively more autonomy as they improved clinically over time and garnered more trust from attendings, which could bias the results in ways we were unable to control. Furthermore, due to the scope of the study and variability in how ACGME cases were logged between training programs, we did not collect trainee case logs prior to the study, limiting the ability to account for background experience in the data analysis.

## Conclusions

OPIs expressing active time and path length indicate low degrees of autonomy in alignment with acknowledged national trends. Autonomy was associated with a slight increase in EBL but not operative time or cost, indicating that trainee experience may not compromise overall operative efficiency. Altogether, OPIs may offer a valuable surrogate of operative autonomy as surgical education shifts towards competency–based milestones. Further investigation is needed to clarify how OPIs relate to clinical outcomes and how to best integrate their use into formal curriculum.

## Supplementary Information

Below is the link to the electronic supplementary material.

Supplementary Material 1

Supplementary Material 2

## Data Availability

All data supporting the findings of this study is stored in an institutional repository which is not publicly available for patient privacy. De-identified data and analyses may be provided upon request and with permission of the Ohio State University data sharing policies.

## Abbreviations

ACGME: Accreditation Council for Graduate Medical Education
AT: Active Time
EBL: Estimated Blood Loss
EPA: Entrustable Professional Activity
IDR: Intuitive Data Recorder
OPI: Objective Performance Indicator(s)
PL: Path Length
STS: Society of Thoracic Surgeons
