# Supplementary material for: Measurement of trainee autonomy in robotic-assisted lung resections using objective performance indicators
Source: J Robot Surg. 2026 Oct 5;20(1):1057. doi: 10.1007/s11701-026-03622-z (PMC13638706; doi:10.1007/s11701-026-03622-z)
Supplement: Supplementary file 1 [file 11701_2026_3622_MOESM1_ESM.docx]

| PGY level | n (%) |
| --- | --- |
| Junior Residents | **17 ( 29%)** |
| PGY-1 | 4 (23%) |
| PGY-2 | 2 (12%) |
| PGY-3 | 11 (65%) |
| Training Pathway |  |
| General Surgery Resident | 15 (88%) |
| Integrated Thoracic Surgery Resident | 2 (12%) |
|  |  |
| Senior Residents | **42 (71%)** |
| PGY-6 | 10 (24%) |
| PGY-7 | 32 (76%) |
| Training Pathway |  |
| Thoracic Surgery Fellow | 38 (90%) |
| Acute Care Surgery Fellow | 4 (10%) |
| Supplemental Table 1. Resident training year and pathway for individual cases. PGY = post-graduate year. Middle-level residents (PGY4-5) did not rotate on the thoracic surgery service during the study period. | |

**Supplemental Table 1. Resident Training Year and Pathway for Individual Cases**
