# Supplementary material for: Measurement of trainee autonomy in robotic-assisted lung resections using objective performance indicators
Source: J Robot Surg. 2026 Oct 5;20(1):1057. doi: 10.1007/s11701-026-03622-z (PMC13638706; doi:10.1007/s11701-026-03622-z)
Supplement: Supplementary file 2 [file 11701_2026_3622_MOESM2_ESM.docx]

**Supplemental Table 2: Univariable Associations of Autonomy with Perioperative Outcomes**

| Perioperative Outcomes | Active Time | | Path Length | |
| --- | --- | --- | --- | --- |
|  | **β (95% CI)** | **P-value** | **β (95% CI)** | **P-value** |
| OR time (min) | -33.56 (-90.64 to 23.52) | 0.254 | -44.39 (-124.23 to 35.46) | 0.281 |
| OR cost ($) | -22441.00 (-46362.80 to 1480.80) | 0.071 | -30750.00 (-64215.04 to 2715.04) | 0.077 |
| Chest tube duration (days) | 0.02 (-4.00 to 4.04) | 0.993 | -0.18 (-5.78 to 5.42) | 0.950 |
| Length of stay (days) | -0.58 (-3.00 to 1.83) | 0.636 | -0.57 (-3.93 to 2.80) | 0.742 |
| Estimated blood loss (mL) | 18.01 (0.66 to 35.36) | **0.047** | 26.52 (2.38 to 50.65) | **0.036** |
| Supplemental Table 2. Univariable linear regression analysis of active time and path length with perioperative outcomes. OR = operating room. | | | | |
